# Thickness of the Schneiderian membrane and its correlation with anatomical structures and demographic parameters using CBCT tomography: a retrospective study

**DOI:** 10.1186/s40729-018-0143-5

**Published:** 2018-10-19

**Authors:** Demos Kalyvas, Andreas Kapsalas, Sofia Paikou, Konstantinos Tsiklakis

**Affiliations:** 1 Department of Oral and Maxillofacial Surgery, School of Dentistry, National and Kapodistrian University of Athens, Greece, Thivon 2 str, 11527 Athens, Greece; 2 Oral Diagnosis & Radiology Clinic, School of Dentistry, National and Kapodistrian University of Athens, Greece, Thivon 2 str, 11527 Athens, Greece

**Keywords:** Schneiderian membrane, Sinus, Thickness, Width, CBCT

## Abstract

**Background:**

The aims of the present study were to determine the thickness of the Schneiderian membrane and identify the width of the maxillary sinus, which is indicated by the buccal and lingual walls of the sinus angle between. Furthermore, to investigate the possibility of a correlation between the aforementioned structures and also other anatomical and demographic parameters using CBCTs for dental implant surgical planning.

**Methods:**

The study included CBCT images of 76 consecutive patients with field-of-view 15 × 12 or 12 × 8cm. Reformatted cross-sectional CBCT slices were analyzed with regard to the thickness of the Schneiderian membrane designated by the medial and the lateral walls of the sinus, in three different standardized points of reference. Age, gender, and position of the measurement were evaluated as factors that could influence the dimensions of the anatomical structures, using univariate and multivariate random effects regression model.

**Results:**

The mean thickness of the Schneiderian membrane was 1.60 ± 1.20 mm. The average thickness revealed now differentiation by age (*p* = 0.878), whereas gender seemed to influence the mean thickness (*p* = 0.010). Also, the thickness of the Schneiderian membrane increased from medial to distal (*p* = 0.060). The mean value of the angle designated by buccal and lingual walls of the sinus was 73.41 ± 6.89 °. The angle measurements revealed no correlation with age, but a tendency towards lower mean angles in females (2.5 ° on average, *p* = 0.097). According to the anatomical position of the measurement, a differentiation was also detected. No correlation between thickness of the Schneiderian membrane and the angle of the walls of the sinus was concluded (*p* = 0.662).

**Conclusions:**

This study demonstrated that the thickness of the Schneiderian membrane and the width of the maxillary sinus can only be affected by gender and anatomical position, but not by the age of the patient.

## Background

The maxillary sinus is the largest of the paranasal air-filled spaces, and it develops firstly in utero [1, 2]. Anatomically, the maxillary sinus is a pyramid-shaped cavity located in the facial skull with a mean volume of 12.5 mL (min 5 mL and max 22 mL) [2–6]. The size, the shape, and the wall thickness of every maxillary sinus not only vary among the population, but also between the two sides of an individual skull [6].

The Schneiderian membrane is the mucous membrane that covers the inner part of the maxillary sinus cavity [7]. Histologically, it consists of an overlaid periosteum with a thin layer of a pseudo-stratified ciliated epithelium and highly vascularized connective tissue [1, 7]. According to Kim et al. 2009, it has been proved that mesenchymal stem cells from the sinus membrane have an ability of bone formation, which plays a vital role in sinus floor elevation procedures [8]. Many studies have measured the thickness of the Schneiderian membrane using different methods such as cadaver examinations, CTs, and CBCTs. Normally, the thickness of the Schneiderian membrane is approximately 1 mm [6, 9]. However, in everyday clinical practice, mucosal thickening of the maxillary sinus is a common radiographic finding in asymptomatic patients; therefore, mucosal lining of more than 4 mm is considered to be pathological [6, 9].

When planning any surgical treatment for the maxilla that includes the posterior region, not only the dimensions and abilities of the Schneiderian membrane, but also the anatomical variations of the maxillary sinus are very significant for every clinician. Cone-beam computed tomography provides essential three-dimensional information regarding the inner part of the maxillary sinus in order to increase the success rate of every surgical procedure and, simultaneously, in order to limit the intra- and post-operative complications.

In the international literature, there is a limited number of studies that quantify the dimensions of the Schneiderian membrane using CBCTs. Therefore, the aim of the present retrospective study is to measure the thickness of the Schneiderian membrane and to identify the width of the maxillary sinus, which is indicated by the angle between the buccal and lingual walls of the sinus in a given height, using CBCT imaging. Furthermore, the present study detects possible correlations between the aforementioned factors and also between each of these factors with anatomical locations and demographic parameters.

## Methods

### Patient selection

The study sample included 76 patients, of which 39 were females and 37 were males. In total, 120 sinuses (44 both left- and right-sided, 21 right-sided, and 11 left-sided) were evaluated as suitable for the present study and were measured. The total sample was classified in four age groups (below 45 years, 45–54 years, 54–64 years, and over 65 years of age). The mean age value of the sample is 58 years (ranging from 19 to 65 years of age).

All patients were Greek adults, and all CBCT examinations were performed to evaluate the posterior maxilla for future implant surgery. All patients were either patients of our clinic or had been referred to our clinic by their private dentist for implant or other pre-implant surgery. The minimum acceptable alveolar process was 5 mm. Patients with previous implant therapy or/and maxillary sinus augmentation in their history as well as patients with active periodontal disease, cysts, polyps, sinusitis, allergic rhinitis, or other pathological entities in their maxillary sinuses were excluded from our study. Other exclusion criteria were the presence of systematic diseases impacting the metabolism and quality of the bones in patients’ history, such as thyroid disease, hyperparathyroidism, diabetes, chronic renal disease, osteoporosis, and no development or acquired craniofacial or neuromuscular deformities. None of the patients previously or currently received medication including vitamin D, human growing hormone (HGH), or bisphosphonates. All the collected data were anonymous.

### Imaging procedure

The CBCT images were obtained with NewTom VGI Tomograph (NewTom, Verona, Italy) with a voxel size of 0.3 mm. Operating parameters were set at 5.28 mA, 110 kV, exposure time 3.60 s, and field of view (FOV) of 15 × 12 or 12 × 8 cm. Panoramic reformatted images with thickness of 1 mm were chosen for the present study.

All measurements were performed in millimeters using the ruler contained in the NNT Viewer®.

**Fig. 1:**
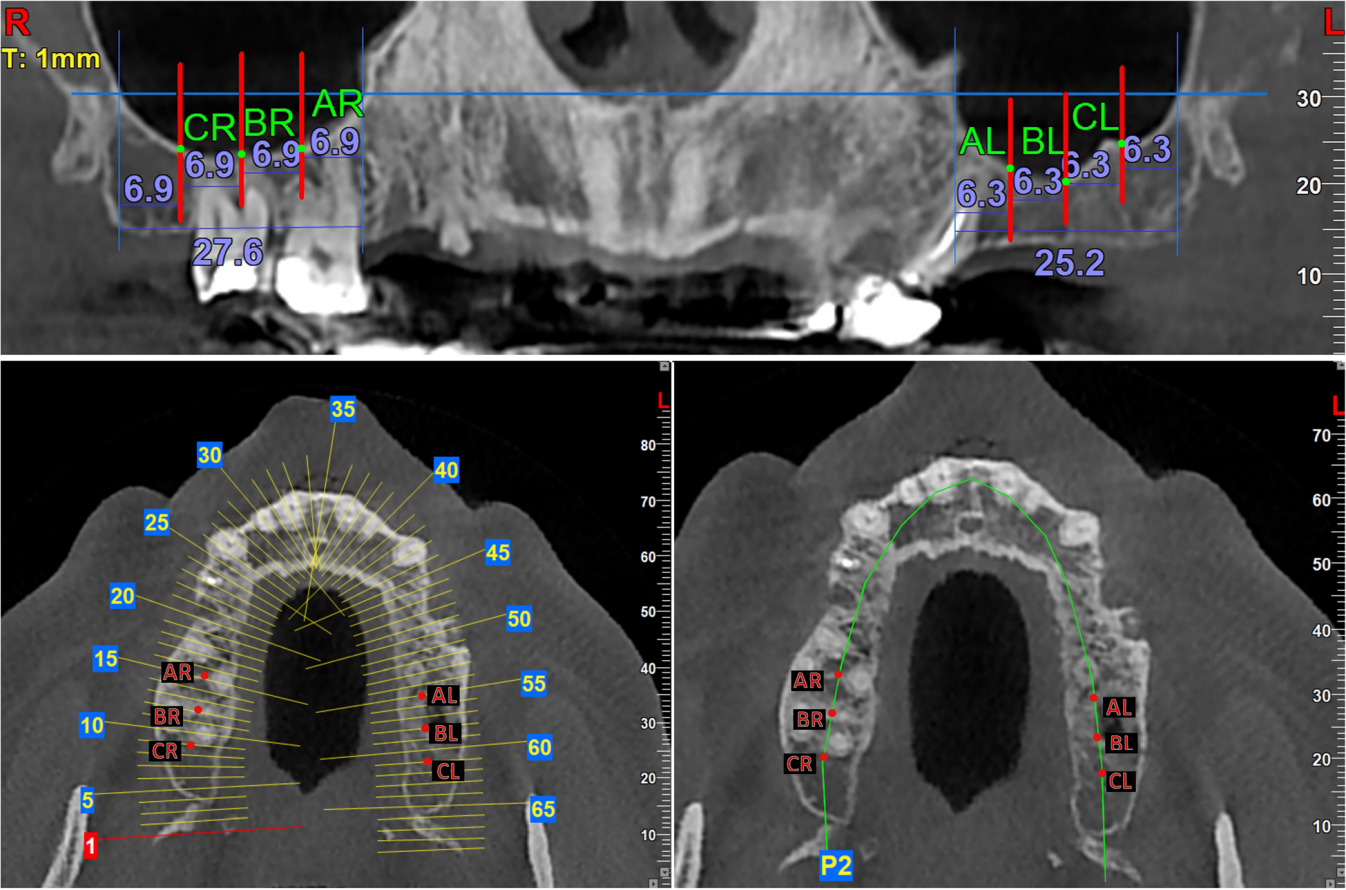
Demonstration of the method used in the panoramic image to divide the sinus in four equal parts and find three fixed points for the measurements. Also, these fixed points in the horizontal plane with and without sections

**Fig. 2:**
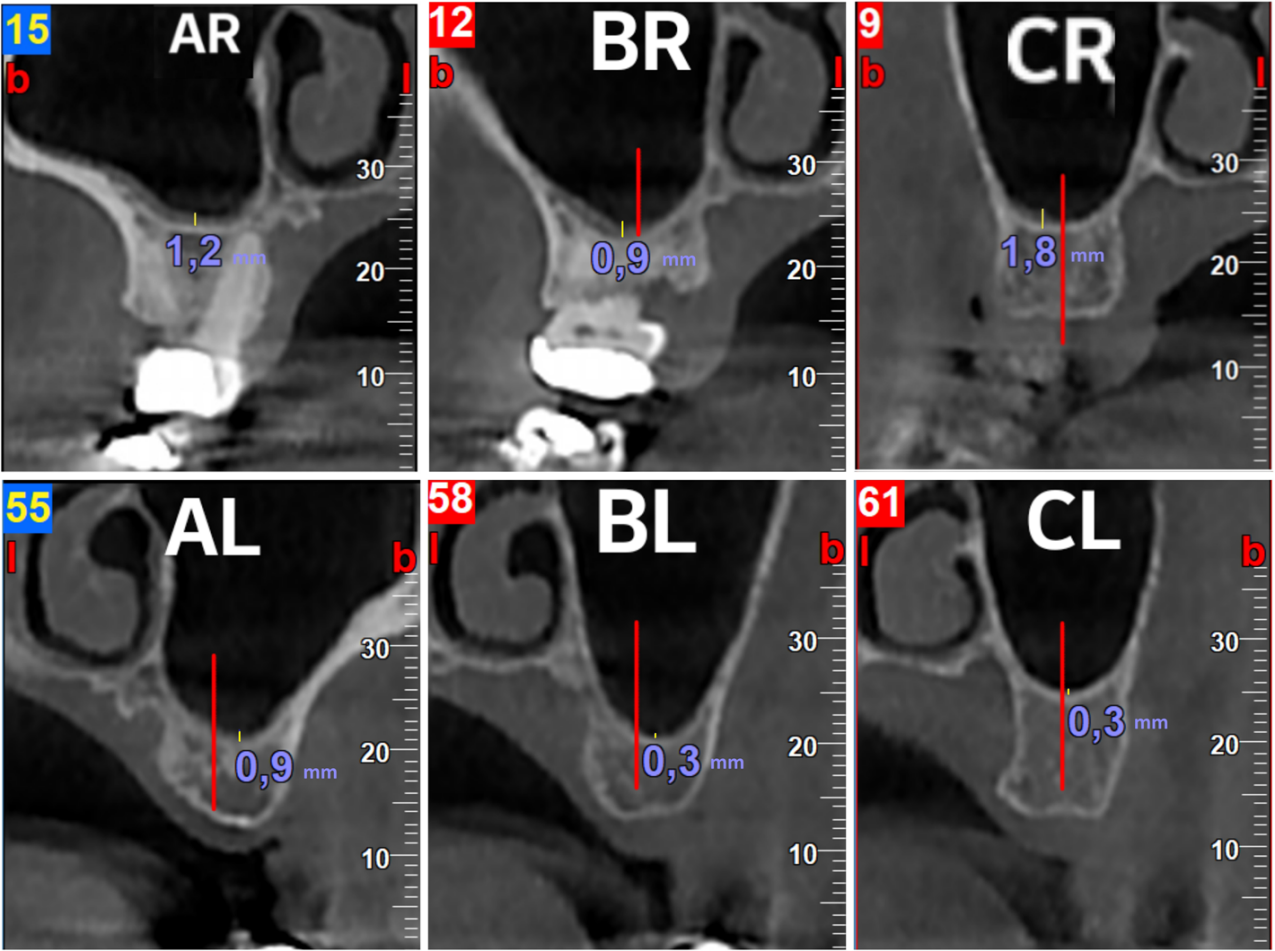
Demonstration of the method used to measure the thickness of the Schneiderian membrane in the cross-sectional images for each of the three fixed points

The distance between the medial and the lateral walls of the maxillary sinus in the panoramic reformatted images was divided in four equal segments. Thus, three points were created; AR, BR, and CR for the right sinus and AL, BL, and CL for the left sinus (Fig. 1). For each of the aforementioned points, a cross section of 1 mm thickness was performed in the middle of the alveolar bone. In the cross-sectional images, the thickness of the Schneiderian membrane was measured in the deepest point of the sinus floor (point D). Thus, three different measurements were performed for each sinus (Fig. 2).

**Fig. 3:**
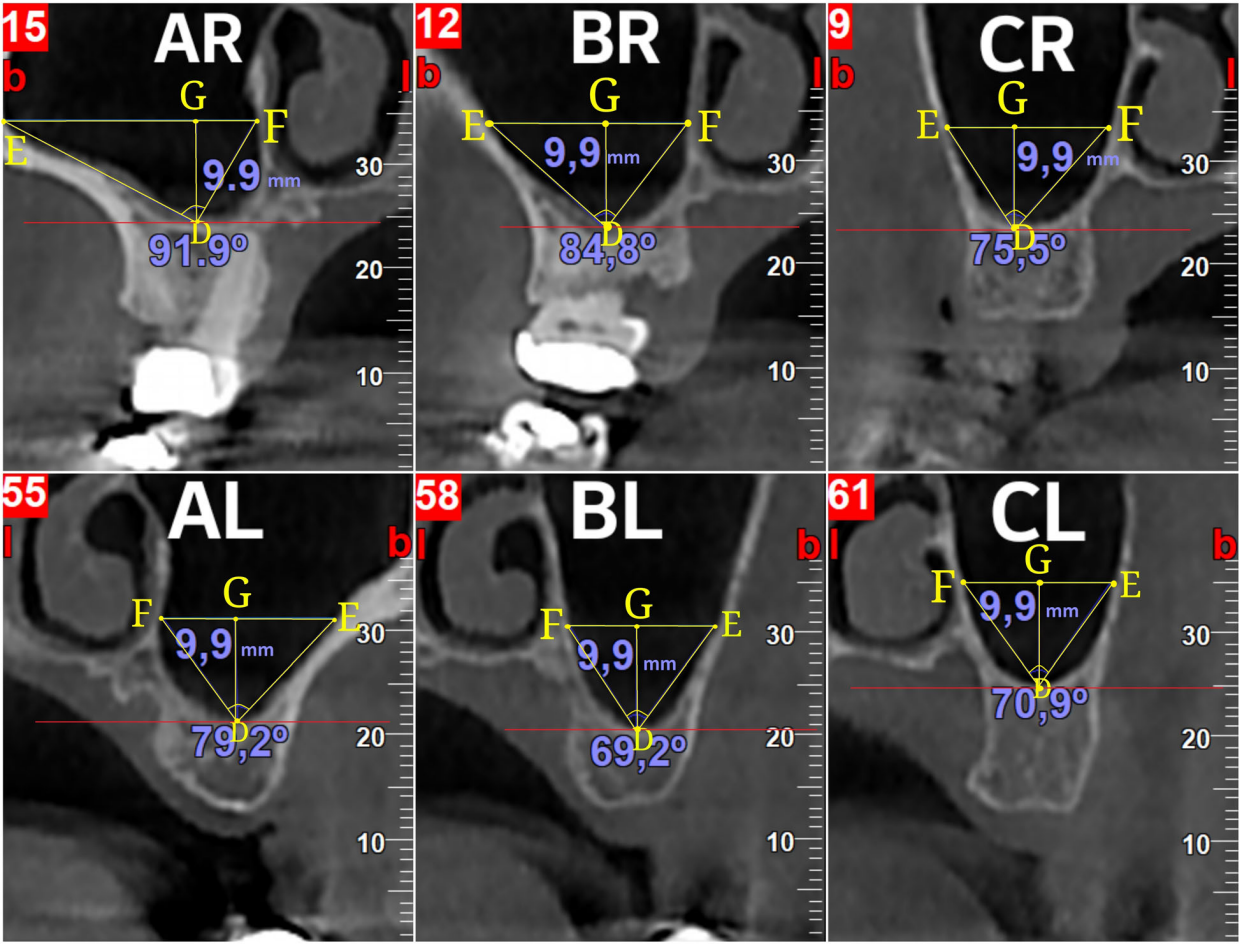
Demonstration of the method used to measure the angle designated by the buccal and lingual walls of the sinus angle for each of the three fixed points in a given height

These three cross-sectional images, in which the thickness of the Schneiderian membrane was previously measured, were also used for the measurement of the angle of the maxillary sinus. A segment DG (point D is the deepest point of the floor of the maxillary sinus) is created, vertical to the horizontal plane with stable length equal to 9.9 mm. The mean of 9.9 mm was chosen, because of a limitation of the NNT® software’s limitation. A linear segment EGF (point E is the point where the segment EGF intersects with the buccal wall of the maxillary sinus, and point F is the point where the segment EGF intersects with the lingual wall of the maxillary sinus) is created which vertical to segment DG and parallel to the horizontal plane. The points D, E, and F designate a triangle named DEF. The angle which is measured is EDF (Fig. 3).

Using the built-in ruler of the NNT Viewer®, we measured the angle EDF for each cross-sectional image created by the aforementioned method. This angle indicates the width of the maxillary sinus in each location. As this angle increases, so does the width of the maxillary sinus.

According to the value of this angle, the sinus width was classified in one of the three following groups (small-, moderate-, and large-sized sinus):

- Angle ≤ 65^o^: small
- 65^o^ < angle < 80^o^: moderate
- Angle ≥ 80^o^: large

### Evaluation of the images

The CBCT images were initially evaluated by an oral surgeon and then they were re-evaluated and measured by two experienced dentists who were involved in the patients’ treatment and follow-up. In case of any disagreement, both parties re-evaluated and remeasured the related CBCT imaging.

### Statistical analysis

All analyses were performed using Stata version 13.1 (Stata Corp., TX, USA).

## Results

The mean value of the overall average thickness is 1.60 ± 1.20 mm (males 1.95 ± 1.28 mm and females 1.24 ± 1.02 mm) (Table 1).

**Table 1:** Sinuses’ thickness by gender and overall

	Sex			
	Male	Female	Overall	
	Mean (SD)	Mean (SD)	Mean (SD)	*p* value
Thickness (mm)-AR	2.06 (1.54)	1.36 (2.09)	1.73 (1.84)	0.131
Thickness (mm)-BR	1.77 (1.43)	1.00 (0.82)	1.40 (1.23)	0.010
Thickness (mm)-CR	1.54 (1.06)	0.77 (0.61)	1.17 (0.95)	0.001
Thickness (mm)-AL	2.13 (1.96)	1.64 (2.54)	1.88 (2.28)	0.431
Thickness (mm)-BL	2.02 (2.09)	1.46 (1.08)	1.72 (1.64)	0.210
Thickness (mm)-CL	1.72 (1.58)	1.26 (1.18)	1.48 (1.39)	0.226
Average thickness (mm)—all points	1.95 (1.28)	1.24 (1.02)	*1.60 (1.20)*	*0.010*

Significant entries are in italic

The average thickness of the membrane also showed no tendency for differentiation by age group (*p* = 0.878) (Table 2).

**Table 2:** Average thickness by age group

	Age (years)					
	< 45	45–54	55–64	65+	Overall	
	*N* (%)	*N* (%)	*N* (%)	*N* (%)	*N* (%)	*p* value
Total	14 (100.0)	14 (100.0)	28 (100.0)	20 (100.0)	76 (100.0)	
	Mean (SD)	Mean (SD)	Mean (SD)	Mean (SD)	Mean (SD)	*p* value
Average thickness (mm)—overall	1.55 (1.21)	1.86 (1.57)	1.61 (1.25)	1.45 (0.86)	1.60 (1.20)	0.878

The statistical analysis also shows a clear tendency towards lower values when checking from point AR to point CR and from point AL to point CL, which means that the average thickness of the membrane increases from medial to distal. (*p* = 0.060) (Table 3).

**Table 3:** Thickness by point of measurement (all measurements)

	Position				
	1 (AR/AL)	2(BR/BL)	3 (CR/CL)	Overall	
	*N* (%)	*N* (%)	*N* (%)	*N* (%)	*p* value
Total	120 (100.0)	120 (100.0)	120 (100.0)	360 (100.0)	
	Mean (SD)	Mean (SD)	Mean (SD)	Mean (SD)	*p* value
Thickness (mm)	1.8 (2.0)	1.6 (1.4)	1.3 (1.2)	1.6 (1.6)	*0.060*

The average mean value of the angle of the maxillary sinus in cross sections was 73.41 ± 6.89°, revealing high prevalence of moderate-shaped sinuses (average mean angle value 74.65 ± 8.08 for the male group and 72.11 ± 5.17 for the female group) (Table 4).

**Table 4:** Sinuses’ angles by gender and overall

	Gender			
	Male	Female	Overall	
	*N* (%)	*N* (%)	*N* (%)	*p* value
Sinuses measured	21 (53.8)	23 (62.2)	44 (57.9)	
	Mean (SD)	Mean (SD)	Mean (SD)	*p* value
Angle (°)-AR	71.79 (16.56)	64.13 (12.09)	68.14 (14.99)	0.039
Angle (°)-BR	80.34 (9.56)	78.60 (5.63)	79.51 (7.92)	0.380
Angle (°)-CR	71.06 (6.82)	71.64 (6.02)	71.34 (6.41)	0.719
Angle (°)-AL	73.87 (14.70)	69.08 (12.43)	71.34 (13.64)	0.197
Angle (°)-BL	80.34 (8.21)	79.25 (7.83)	79.77 (7.96)	0.616
Angle (°)-CL	72.33 (8.15)	72.59 (6.04)	72.47 (7.05)	0.890
Average angle (°)—all points	74.65 (8.08)	72.11 (5.17)	73.41 (6.89)	0.109

The angle of the sinus also shows no association with age (Table 5).

**Table 5:** Angle of the walls of the sinus by age groups

	Age (years)					
	< 45	45–54	55–64	65+	Overall	
	Mean (SD)	Mean (SD)	Mean (SD)	Mean (SD)	Mean (SD)	*p* value
Angle (°)-AR	70.05 (17.57)	68.92 (15.67)	67.43 (12.05)	67.29 (17.30)	68.14 (14.99)	*0.958*
Angle (°)-BR	80.63 (8.11)	78.50 (10.66)	78.58 (5.70)	80.74 (8.43)	79.51 (7.92)	*0.766*
Angle (°)-CR	70.80 (7.38)	71.31 (7.73)	69.57 (5.31)	73.93 (5.69)	71.34 (6.41)	*0.190*
Angle (°)-AL	76.95 (9.55)	67.30 (12.39)	73.75 (14.11)	66.01 (14.75)	71.34 (13.64)	*0.136*
Angle (°)-BL	79.35 (6.37)	82.81 (10.87)	79.61 (7.65)	78.67 (8.11)	79.77 (7.96)	*0.694*
Angle (°)-CL	73.71 (8.66)	74.44 (4.38)	71.27 (7.52)	72.19 (6.50)	72.47 (7.05)	*0.671*

On the contrary, it was proven that there is a tendency towards lower angle values in the female group (2.5°on average, *p* = 0.097). It was also shown that at the points BR and BL, the angles were statistically significantly higher than those at the points AR and AL, of the order of 10 ° (*p* < 0.001), but at the points CR and CL the difference is of 2.2 ° (*p* = 0.051). No association between thickness of the membrane and the angle (width) of the current position in the sinus was detected (*p* = 0.662) (Table 6).

**Table 6:** Results from a multivariable random effects regression model of all angles on age (in groups) gender and point of measurement

Factor	Difference	95% CI	*p* value
Age (years)			
< 45^a^	0		
45–54	− 0.9	(− 5.7, 3.9)	0.724
55–64	− 1.4	(− 5.5, 2.8)	0.520
65+	− 0.6	(− 5.0, 3.9)	0.806
Gender			
Male^a^	0		
Female	− 2.5	(− 5.4, 0.5)	0.097
Position			
1 (AR/AL)^a^	0		
2(BR/BL)	10.0	(7.8, 12.3)	< 0.001
3 (CR/CL)	2.2	(−0.0, 4.5)	0.051

^a^Reference category

## Discussion

It is very important to pre-operatively evaluate the thickness of the Schneiderian membrane to plan the surgical procedure in the region that involves the membrane, such as a sinus lift augmentation, which increases the possibility of membrane perforation or other complications.

The present study assumed that the average thickness of the Schneiderian membrane is 1.60 ± 1.20 mm.

There are many studies which estimate the average thickness of the mucosa of the maxillary sinus, some of which have used CBCTs while others have not. These studies are presented in Table 7. These values are not completely different to the results of the present study. From these studies, we can conclude that, clearly, there is variability regarding the thickness of the Schneiderian membrane. This means that a clinician cannot collect information about the thickness of a sinus membrane by patient sex, side, or age.

**Table 7:** All studies measuring the thickness of the Schneiderian membrane [5, 7, 9, 12, 15, 17–25]

Authors	Year of study	Method of study	Results
Tos and Mogesen et al.	1979	Cadavers	0.3–0.8 mm
Aimetti et al.	2008	Endoscopically	0.97 ± 0.36 mm
Pommer et al.	2009	Cadavers	0.09 ± 0.045 mm
Janner et al.	2010	CBCTs	2.16–3.11 mm (mid-sagittal regions) and 0.9–1.84 mm (lateral-median regions)
Pazera et al.	2010	CBCTs	1.58 mm (95% CI 1.17–1.98)
Cakur et al.	2011	CBCTs	0.5 ± 0.49 mm
Pommer et al.	2012	CTs	0.8–1.99 mm
Anduze-Acher et al	2012	CTs	1.99 ± 2.10 mm
Zheng-Ze Guo et al.	2015	CBCTs	1.93 ± 2.00 mm
Shih-Cheng Wen et al.	2015	CBCTs	1.78 ± 1.99 mm
Yen-Hua Lin et al.	2015	CBCTs	1.32 ± 0.87 mm
Insua et al.	2017	Cadavers	1.36 ± 0.42 mm
Lozano-Carrascal et al.	2017	CBCTs	1.82 ± 1.59 mm
Talo Jildirim et al.	2017	CBCTs	4.19 ± 5.84 mm
Present study	2017	CBCTs	1.60 ± 1.20 mm

In this study, it was detected that 27.5% of the measurements made (99 out of 360) were more than 2 mm. However, all X-ray examinations that showed a visually detectable pathology were excluded from our study. For this reason, our study cannot conclude that the limit of 2 mm is an accurate limit to evaluate a sinus membrane as healthy or pathological. The limit of 2 mm as a criterion is used by Shanbhag et al. 2014, Ji-Young Yoo et al. 2011, Janner et al. 2011, and Cagici et al. 2008 [10–13]. On the contrary, Eggesbo et al. 2006 and Cakur et al. 2011 set the limit of 4 mm as a safe threshold to evaluate a sinus membrane as pathological [9, 14]. Furthermore, Lozano-Carrascal et al. 2017 accept a threshold of 3 mm of membrane thickness for pathology existence [15]. In our study, the membrane thickness of 6.94% of our sample was over 4 mm. For this reason, we can accept a thickness over 4 mm as a safer limit to evaluate if there is a pathology in the sinus or not.

Comparing the thickness of the membrane between the two genders, males seem to have thicker membranes than females. Vallo et al. 2010, Janner et al. 2011, Ji-Young Yoo et al. 2011, Cakur et al. 2013, and Jildirim et al. 2017 [9, 11, 12, 16, 17] also come to this conclusion. Our study assumed that this difference is of the order of 40%. On the contrary, Pazera et al. 2010 concluded that there is no significant difference between thickness of the mucosa and gender (*p* = 0.294) [18].

The aforementioned differences can be attributed to the fact that every study has evaluated different populations and has also set different criteria in the selection of their sample.

In our study, there is no statistically significant influence when comparing the thickness of the mucosa and age (*p* = 0.878). Janner et al. 2010, Vallo et al. 2010, and Jildirim et al. 2017 also come in accordance with our results [12, 16, 17]. The age factor does not seem to affect the anatomical characteristics of the sinus. Unlike a state of inflammation, allergy, smoking of the patient does not affect the sinus thickness or wideness [6].

As regards the width of the maxillary sinus, the mean angle value was found to be 73.4 ± 6.9 °. It seems that there is a tendency towards greater angle values in the male group, but the difference is not statistically significant in order to conclude that wider angles and widths exist in male patients. The aforementioned results can be also associated with the results of the study of Gosau et al. 2009, which showed that female sinuses tend to have lower average volume values than male sinuses [4]. The study of Lozano-Carrascal et al. 2017 also measured the same angle but only in the region of the upper first molar and found a mean value of 73.39 ± 15.23 ° [15]. The present study is more accurate because the mean angle results from three different measurements taken from three different points of the maxillary sinus.

In the present study, it was also concluded that the width of the sinus increases from mesial to distal. Male sinuses had higher prevalence of high angle values compared to female sinuses, but the majority of angle values and widths was characterized as moderate.

In an attempt to correlate the membrane biotype regarding thickness with the sinus width, it was proven that there is no correlation between the thickness and width of the sinus (*p* = 0.695).

## Conclusions

In conclusion, the present study demonstrated that male patients tend to have a thicker membrane than female patients. The angles of the sinus seemed to increase in width from mesial to distal, and they have no significant correlation with any of our parameters. Thickness of the mucosa and width of the maxillary sinus did not seem to correlate. Future studies including larger groups of participants should be necessarily conducted in order to establish additional possible correlations between the anatomical structures of the maxillary sinus and their variations.
